# Lateral meniscus autograft transplantation using hamstring tendon with a sandwiched bone marrow - derived fibrin clot: A case report

**DOI:** 10.1016/j.ijscr.2023.108444

**Published:** 2023-06-30

**Authors:** Ken Iida, Yusuke Hashimoto, Kazuya Nishino, Yohei Nishida, Hiroaki Nakamura

**Affiliations:** a Department of Orthopaedic Surgery, Osaka Metropolitan University Graduate School of Medicine, Osaka, Japan; b Department of Orthopaedic Surgery, Saiseikai Nakatsu Hospital, Osaka, Japan

**Keywords:** Meniscal transplantation, Tendon autograft, Bone marrow - derived fibrin clot, T2 mapping, Case report

## Abstract

**Introduction and importance:**

Tendon autograft is a durable solution for the sub/total meniscus; however it is still considered a temporary solution.

**Case presentation:**

We report the case of a 17-year-old woman with history of subtotal lateral meniscectomy performed 6 years ago. We treated her with lateral meniscus autograft transplantation using a hamstring tendon with a sandwiched bone marrow aspirate (BMA)-derived fibrin clot. T2 relaxation times of the anterior and posterior horns of both menisci and of the cartilage were assessed.

**Clinical discussion:**

Lateral meniscus autograft transplantation using a hamstring tendon with a sandwiched BMA clot improved clinical and radiographic outcomes at the 24-month follow-up. These findings suggest that the lateral meniscus autograft transplantation using a hamstring tendon with a sandwiched BMA clot transformed into a meniscus-like tissue and resulted in preservation of the articular cartilage.

**Conclusion:**

Lateral meniscus autograft transplantation using a hamstring tendon with a sandwiched BMA clot can function as a meniscal transplant after total or subtotal meniscectomy in young patients.

## Highlights

• Tendon autograft is a durable solution for the sub/total meniscus, it is still considered a temporary solution.
• A 17-year-old woman who underwent subtotal lateral meniscectomy and was later treated her with lateral meniscus autograft transplantation using a hamstring tendon with a sandwiched bone marrow aspirate-derived fibrin clot (BMA clot).
• Lateral meniscus autograft transplantation using a hamstring tendon with a sandwiched BMA clot is a viable option for meniscal transplantation after total or subtotal meniscectomy in young patients.

## 1 Introduction

Meniscus injuries are common, and removal of meniscal tissue leads to a significant increase in the risk of loss of knee function and development of osteoarthritis [1]. Treatment of the knee after meniscectomy remains difficult, especially in young patients. For certain symptomatic patients who have previously undergone total or subtotal meniscectomy, meniscal transplantation may be considered as an alternative management option [2]. Meniscus transplant aims to reduce pain and swelling, prevent or slow the progression of osteoarthritis, and improve the stability of the knee joint. Although this procedure provides good clinical results, in some countries it may be difficult to perform allogeneic transplants for legal and/or ethical reasons. Another option could be a distal femoral osteotomy. However, little is known about the results of this procedure for good functional recovery in young patients [3]. Kohn et al. conducted an animal study to investigate the chondroprotective effect of using patellar tendon autograft as a meniscus graft after meniscectomy [4]. Although a tendon autograft is a durable solution for the sub/total meniscus, it is still considered a temporary solution.

In this case report, we present a patient who underwent subtotal lateral meniscectomy and was later treated with lateral meniscus autograft transplantation using a hamstring tendon with a sandwiched bone marrow aspirate-derived fibrin clot (BMA clot). The work has been reported in line with the SCARE criteria. [5]

## 2 Presentation of case

**Fig. 1:**
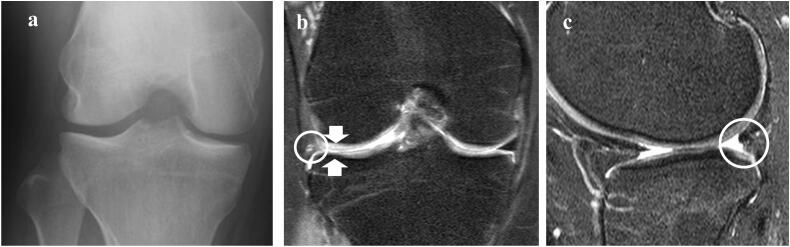
Preoperative images. **a** Rosenberg view X-ray of the lateral compartment shows a lateral joint space width of 2.9 mm. **b** Coronal magnetic resonance imaging (MRI) demonstrates the near complete disappearance of the middle part of the lateral meniscus (white circle) along with cartilage damage (white arrow). **c** Sagittal MRI shows that the posterior part of the lateral meniscus has almost disappeared (white circle).

The patient was a 17-year-old woman who presented with right knee pain. She had undergone subtotal lateral meniscectomy 6 years ago at another hospital. For a while, she was able to exercise without any symptoms; however, she started to experience pain on the lateral side of the knee with swelling 2 years after the operation. The preoperative Tegner activity score was 5. Physical examination revealed swelling and tenderness of the lateral knee joint. The range of motion of her knee joint was 0°–130°. An X-ray with the Rosenberg view (standing PA view with 45° knee flexion) revealed the width of the lateral joint space as 2.9 mm (Fig. 1a). Magnetic resonance imaging (MRI) revealed minor cartilage defects in the lateral compartment, and the middle and posterior parts of the lateral meniscus had almost diminutive appearance (Fig. 1b, c). Due to the refractory pain and difficulty in performing daily activities, operative treatment with lateral meniscus autograft transplantation using a hamstring tendon with a sandwiched BMA clot was chosen.

**Fig. 2:**
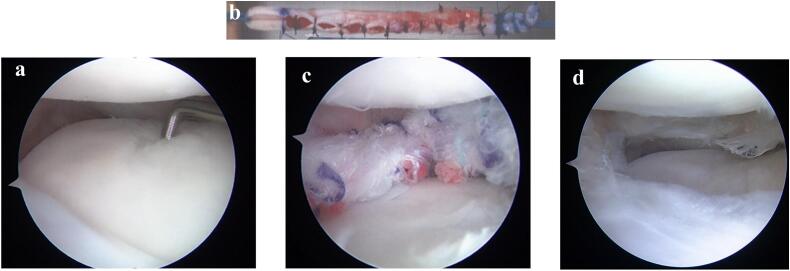
Arthroscopic findings. **a** In the lateral compartment, the lateral meniscus from the midbody to the posterior part has almost diminutive appearance, and minor chondral defects (ICRS grade 3) can be seen in both the tibial plateau and the femoral condyle. **b** The hamstring tendon is folded over the distal round part creating a double-stranded loop with the sandwich BMA clot. **c** Lateral meniscus transplant in position. **d** Partial coverage with white fibrocartilage classified as ICRS grade 2 is visible during second-look arthroscopy.

Standard arthroscopic examinations were performed from the anteromedial and anterolateral portals. In the lateral compartment, it was observed that the lateral meniscus in the mid to posterior region had almost completely disappeared, and massive chondral defects with International Cartilage Repair Society (ICRS) grade 3 were found on both the tibial plateau and the femoral condyle (Fig. 2a). To prepare the BMA clots, 10 mL of BMA was collected from the lateral side of the intercondylar notch of the knee joint using an 11-gauge bone marrow needle, positioned in flexion without a tourniquet [6]. The BMA was placed in a sterile glass dish and stirred slowly with a glass rod to promote blood clotting. Clots began to form in the dish within 5–10 min and were separated from the serum after clotting. The clot was picked up from the dish and placed on a gauze. The semitendinosus was grasped and harvested using an open stripper. After harvesting the hamstring, the graft was cleaned of any muscle tissue, and the proximal flat part of the tendon was folded over the distal round part, creating a double-stranded loop containing the sandwiched BMA clot (Fig. 2b). The anterior and posterior root tunnels were created as close to the anatomical position as possible using the meniscus root guide and flip-cutter (Arthrex®) with dimensions corresponding to the graft size. The length of the graft was 12 cm and diameter was 5.5 mm. The tendon graft was inserted through the accessory portal, and the folded end of the graft was pulled into the posterior root tunnel. Then, a blunt instrument was used to push the graft along the boundary of the capsule. The sutures from the other graft ends were retrieved and pulled down the anterior root tunnel. Inside-out and outside-in sutures with a mechanical insertion device (Meniscal Suture Kit; Stryker) were used around the graft (Fig. 2c). Tibial fixation of the graft was performed using a double spike plate (Meira Co., Ltd.; Nagoya, Japan) with a 6.5-mm screw.

**Fig. 3:**
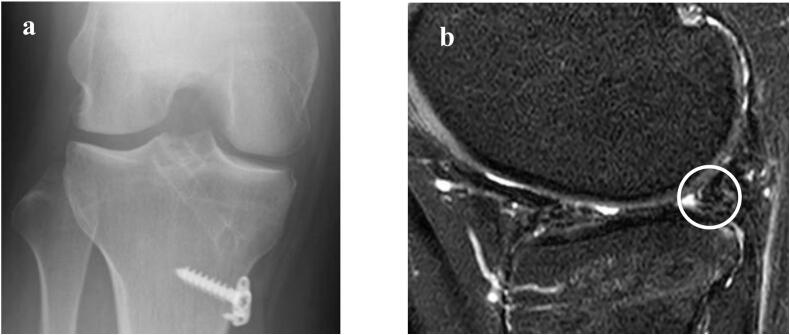
Postoperative imaging at 24 months. **a** Rosenberg view X-ray of the lateral compartment shows lateral joint space widening to 3.7 mm. **b** Sagittal magnetic resonance imaging (MRI) demonstrates the autograft in the posterior part of the lateral meniscus (white circle).

**Fig. 4:**
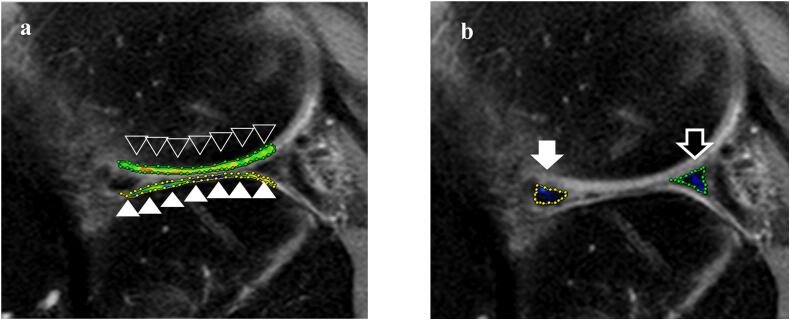
**a** Region of interest (ROI) for the measurement of the T2 relaxation time at the lateral femoral condyle (black arrowhead) and tibial plateau (white arrowhead). **b** The ROI for the measurement of the T2 relaxation time at the anterior horns (white arrow) and posterior horns (black arrow) of the meniscus.

The postoperative rehabilitation protocol for meniscus repair was followed. The patient was immobilized with a knee immobilizer for 1 week postoperatively, and the range of motion of the knee joint was restricted to 0°–90° for the following 3 weeks. A 4-week non-weight-bearing period was prescribed; partial weight-bearing started at 4 weeks postoperatively, full weight-bearing at 8 weeks, jogging at 3 months, and return to previous sports at 6 months. At the 2-year follow-up, she was able to exercise without lateral joint pain and swelling. The postoperative Tegner activity score was 6. The range of motion had improved to 0°–140° which is a 10-degree improvement from her preoperative range of motion, and the McMurray test was negative. The Lysholm score improved from 46 preoperatively to 95 at 2 years after surgery, and the International Knee Documentation Committee score improved from 47.4 preoperatively to 93.5 at 2 years after surgery. The lateral joint space widened from 2.9 mm preoperatively to 3.7 mm at 2 years after surgery (Fig. 3a). Partial coverage with white fibrocartilage was classified as ICRS grade 2 at the time of second-look arthroscopy two years after lateral meniscus autograft transplantation. (Fig. 2d). MRI demonstrated the autograft at the posterior part of lateral meniscus (Fig. 3b). To evaluate the quality of the lateral meniscus autograft and cartilage, quantitative MRI with T2 mapping was performed and T2 relaxation time measurements non-invasively assess early cartilage degeneration reflecting changes of the biochemical composition of the articular cartilage and menisci. [7]. MRI examinations were performed preoperatively and at 3, 6, 12, and 24 months postoperatively with a 3.0-T scanner (Achieva 3.0 T TX; Philips, Best, The Netherlands). The region of interest was determined in the midsagittal plane, reflecting the center of the lateral femoral condyle and lateral tibial plateau. The cartilage compartment corresponded to the region between the anterior and posterior horns of the meniscus, and the meniscus compartment corresponded to the anterior and posterior horns of the meniscus. The T2 relaxation time of each pixel was calculated using Virtual Place Advance software (AZE Ltd., Tokyo, Japan) (Fig. 4).

The T2 relaxation time of the whole anterior and posterior horns of the meniscus demonstrated a decrease at 3, 6, and 12 months after the surgery and T2 relaxation time at 24 months was increased at 12 months after the surgery. Compared to the preoperative time, the T2 relaxation time of the whole lateral femoral condyle increased at 3, 6, and 12 months after the surgery and decreased at 24 months. Compared to the preoperative time, the T2 relaxation time of the whole lateral tibial plateau increased at 3 months after the surgery followed by a decrease at 24 months. Furthermore, the T2 relaxation time of the whole lateral femoral condyle and lateral tibial plateau at 24 months was lower than that of the entire lateral femoral condyle and lateral tibial plateau preoperatively (Table 1).

**Table 1:** Chronological changes in the T2 relaxation time of the lateral femoral condyle, tibial plateau, and meniscus autograft.

	Preoperative	3 Months	6 Months	12 Months	24 Months
Femoral condyle	48.9	50.2	52.5	53.3	48.7
Tibial plateau	45.5	47.6	45.2	44.4	43.2
Anterior horns of the meniscus		40.1	37.2	35	37.9
Posterior horns of the meniscus		37.3	35.1	34.9	37.1

## 3 Discussion

We reported that a patient who had undergone subtotal lateral meniscectomy and was then treated with lateral meniscus autograft transplantation using a hamstring tendon with a sandwiched BMA clot showed good clinical and radiographic outcomes at the 24-month follow-up.

Kohn et al. reported successful results in terms of both healing and cartilage protection in a clinical study using a portion of the quadriceps tendon as a meniscus autograft [3]. The 12-month results were promising, but detailed data were not published. Johnson et al. reported no clinical improvement or preservation of the joint space after lateral meniscal autograft transplantation. However, the patient demonstrated loss of the lateral space of the joint at the time of surgery [8], indicating that there are limitations to the method of isolated meniscus autograft transplantation.

The fibrin clot is expected to act as a structural support for meniscus healing as well as a chemotactic stimulus and mitogenic growth factor [9]. BMA has been identified as an excellent source of cells and growth factors and has been used successfully for bone, cartilage, and soft-tissue healing [10]. Previous report described an improvement in the postoperative clinical results after meniscal repair using a BMA clot for isolated avascular meniscal injury [6]. Given these advantages of a BMA fibrin clot, it is expected to promote transformation of a tendon into meniscus-like tissue more effectively.

New techniques have been applied to T2 mapping in MRI. Many studies have demonstrated the potential of newly developed MRI scans, such as T2 mapping and T1 rho (T1 relaxation time in rotating frames) mapping to reveal changes in the biochemical composition of cartilage tissue [11,12]. T2 mapping may be sensitive to the water and collagen content of the meniscus [13]. Yamasaki et al. reported that T2 relaxation time at the colored meniscal tear line in the healed menisci had significantly decreased postoperatively as compared to that in the incompletely/not healed menisci [14]. Previous report described that there were significant correlations between the cartilage and meniscal T2 relaxation times at 24 months after surgery [15]. Xie et al. claimed that the T2 values of healthy tendons were 30.4 ± 1.0 ms, and the postoperative longitudinal T2 values of the healing site showed a significantly decreasing trend over time [16]. Previous biochemical studies have shown that collagen fibers are aligned parallel to the longitudinal axis of the tendon and that the water concentration decreases during the healing process after repair surgery [17]. In the present case, the T2 relaxation time of the meniscal autograft decreased at 3, 6, and 12 months after surgery and then T2 relaxation time at 24 months was increased at 12 months after the surgery. These results indicate that the process of healing at the meniscus autograft could be reflected by the decreasing trend in the T2 relaxation time, but qualitative of the meniscus autograft worsens after 12 months. Based on the T2 relaxation time of the cartilage, Previous report demonstrated that the T2 relaxation time of the lateral femorotibial joint cartilage increased at 3 and 6 months postoperatively and then had decreased at 12 and 24 months after reshaping surgery for discoid lateral meniscus [18]. These results suggest that the cartilage was stressed during the early postoperative period and recovered later. Furthermore, Park et al. [19] reported that the mean T2 values of the cartilage following meniscus allograft transplantation returned to the baseline levels after 1 year. In the present case, the T2 relaxation time of the whole lateral femoral condyle and lateral tibial plateau at 24 months was lower than that of the entire lateral femoral condyle and lateral tibial plateau preoperatively. These results indicate that the cartilage was stressed during the postoperative period and recovered later. This was reflected as a gradual decrease in the T2 value during the patient's recovery period in both the femoral condyles and tibial plateaus. The meniscus autograft itself may affect recovery from cartilage damage. These findings suggest that the lateral meniscus autograft transplant composed of a hamstring tendon with a sandwiched BMA clot transformed into a meniscus-like tissue and resulted in preservation of the articular cartilage.

## 4 Conclusion

Lateral meniscus autograft transplantation using hamstring tendon with a sandwiched BMA clot resulted in improved clinical and radiographic outcomes at the 24-month follow-up. Lateral meniscus autograft transplantation using a hamstring tendon with a sandwiched BMA clot is a viable option for meniscal transplantation after total or subtotal meniscectomy in young patients.

## Informed consent

The patient's parents guardian for publication of this case report and accompanying images. A copy of the written consent is available for review by the Editor-in-Chief of this journal on request.

## Ethical approval

The study was approved by the Osaka Metropolitan University Graduate School of Medicine ethics committee and the internal review board of our institution (IRB number: 2728 date of approval: 11.30.2013).

## Funding

Not applicable.

## Author contribution

Ken Iida: Conception and design. Drafting of the article.

Yusuke Hashimoto: Conception, and critical revision of the article for important intellectual content.

Yohei Nishida: Interpretation of data.

Kazuya Nishino: Design and revision of the article.

Hiroaki Nakamura: Conception and design, final approval of the article.

All authors read and approved the final manuscript.

## Research registration number

Not applicable. No research study involved.

## Guarantor

The guarantor of this article is Prof. Hiroaki Nakamura.

## Conflict of interest

Not applicable
